# Perspective of drug design with high-performance computing

**DOI:** 10.1093/nsr/nwab105

**Published:** 2021-06-18

**Authors:** Zhe Li, Hui Li, Kunqian Yu, Hai-Bin Luo

**Affiliations:** Guangdong Provincial Key Laboratory of New Drug Design and Evaluation, School of Pharmaceutical Sciences, Sun Yat-Sen University, China; State Key Laboratory of Drug Research, Shanghai Institute of Materia Medica, Chinese Academy of Sciences, China; Shanghai Institute for Advanced Immunochemical Studies, and School of Life Science and Technology, ShanghaiTech University, China; State Key Laboratory of Drug Research, Shanghai Institute of Materia Medica, Chinese Academy of Sciences, China; Guangdong Provincial Key Laboratory of New Drug Design and Evaluation, School of Pharmaceutical Sciences, Sun Yat-Sen University, China; Key Laboratory of Tropical Biological Resources of Ministry of Education, School of Pharmaceutical Sciences, Hainan University, China

## Abstract

The representative applications, recent advances and possible future directions of computational drug design were summarized, aiming to accelerate the drug discovery with the assistance of the fast-developing high-performance computing.

In recent decades, the development of high-performance computing (HPC) continues to follow Moore's law, with many computational methods in drug design experiencing a renaissance. Binding prediction, virtual screening, molecular dynamics simulations and protein folding are crucial applications of computational drug design to accelerate drug discovery (Fig. 1). Reliable prediction of the receptor/ligand binding affinities that determine the pharmacological effects of drugs is the most important application of HPC in drug discovery, and is the main focus of this Perspective.

**Figure 1.**
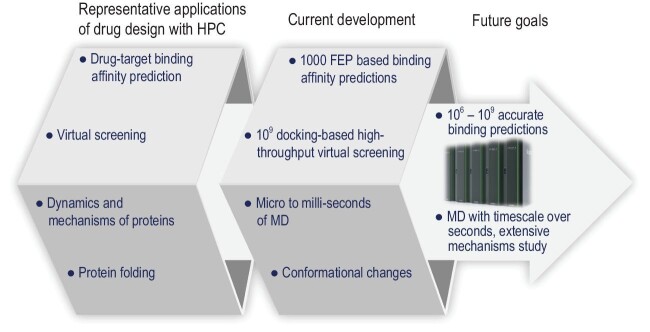
Representative applications of HPC in drug design.

Accurate prediction of receptor/ligand binding affinity remains challenging because of the complexity of the system. There are several methods for predicting the binding affinity, from very fast and approximate empirical scoring functions, to more complicated methods with moderate accuracy such as linear interaction energy and molecular mechanics Poisson—Boltzmann/surface area, to rigorous and accurate but very computationally expensive methods such as free energy perturbation (FEP). Limitations in computational resources set back the theoretical development of the accurate but expensive methods, and also make it difficult to balance accuracy and efficiency for the selection of methods in drug design.

Ultra-high-throughput virtual screening is one of the key demands of drug discovery by HPC. The number of pharmacologically active molecules is extremely large, estimated to be in the order of 10^60^ [1]. The number of purchasable compounds in ZINC database has already reached 230 million, while that of ‘make-on-demand synthesis’ compounds can be 10 billion. For the scoring functions usually implemented in docking methods, ultra-high-throughput virtual screening is now possible by HPC. Although the accuracy of the docking-based virtual screening can be limited, the results are still promising because of the large number of compounds. In 2019, virtual screening of 138 million compounds was performed against D4 receptor, and 30 hits showed sub-micromolar activity including a 180 pM subtype-selective agonist [2]. Recently, an open-source virtual screening platform VirtualFlow was successfully conducted to screen 1.4 billion compounds and identified a potent KEAP1 inhibitor with nanomolar affinity [3].

Accurate binding affinity prediction is another important issue in drug discovery. Docking-based virtual screening showed advantages in hit discovery; however, in further hit-to-lead optimizations where many new compounds could be synthesized, more accurate binding affinity prediction methods are urgently needed to accelerate the process and reduce the cost of synthesis and bioassay validation. For such prediction, extensive MD simulations are needed, because the properties in the macroscopic world, for example free energy, are related to the time average of the corresponding properties in the microscopic system. Although theoretically rigorous, the samples that are important to the accuracy of free energy calculations correspond to the rare events, the lack of sampling of which can be a major source of error. Different enhanced sampling methods have been proposed to overcome the rare events problem, including non-Boltzmann sampling, exchanging configurations, modeling probability distributions, etc. To increase the simulation time, special-purpose supercomputers, such as ANTON2, have been built to perform MD simulations at high speed with considerable stability. Of note, FEP, a representative free energy calculation method, has been successfully used in both absolute and relative binding free energy (ABFE and RBFE) calculations [4–6]. The FEP RBFE method FEP+  was tested by more than 200 ligands with ∼92.4% calculations showing absolute errors within 2 kcal/mol, which was proven to be effective in reducing the false positive rate by further experiments [4]. Relative to FEP RBFE methods, FEP ABFE have a much wider range of applications [5,6] such as scaffold hopping, ligand selectivity study, etc. Recently, a highly potent PDE10 inhibitor with subnanomolar affinity was discovered by FEP ABFE-based hit-to-lead optimizations [5]. However, these accurate binding prediction methods have not been widely applied in real drug discovery works because of high computational costs. With the continuous development of enhanced sampling methods and computational ability, these methods will have more impact on drug discovery in the near future.

Computational drug discovery by HPC has showed greater potential than at any time before in the development of promising agents against COVID-19. Since the outbreak of COVID-19, billion-level ultra-high-throughput virtual screenings were performed to find potential therapeutic agents. The work from LeGrand *et al.* using AutoDock-GPU in the summit supercomputer was shortlisted for the 2020 Gordon Bell Prize. Recently, we also finished a billion-level virtual screening by AutoDock-GPU against RdRp of SARS-CoV-2 in less than 24 hours on a domestic supercomputer. In another work, FEP was used to evaluate the docking-based virtual screening results towards the FDA-approved drug database [6] to give a more accurate ranking in binding affinity.  As a result, 16 hits were identified from 25 selected drugs after bioassay. Among them, dipyridamole showed promising outcomes in the subsequent clinical trials [7]. Additionally, the roles of glycans in modulating the conformational dynamics of the SARS-COV-2 spike protein were also revealed by extensive MD simulations, which provided insight for further drug and vaccine design [8].

Millions or even billions of binding affinity predictions via accurate methods could be one of the ultimate goals for computational drug discovery. However, this would require 10^3^ to 10^6^ times the computational resources of the fastest supercomputer in the world. Currently, combining calculation methods with different speed and accuracy, for example docking-based high-throughput prescreening and FEP-based binding affinity prediction, is an applicable strategy (Fig. 2). Deep learning technologies can also accelerate calculations in drug discovery such as by sampling the Boltzmann distribution, and thus overcome the rare events problems [9]. The convolutional neural networks can be trained to predict the binding affinity from 3D structures [10]. In the foreseeable future, with the development of HPC and technologies such as enhanced sampling and deep learning, more innovative algorithms can be developed, which will further speed up the drug design and discovery.

**Figure 2.**
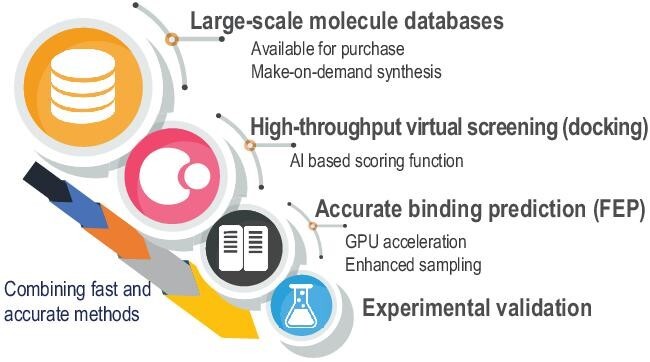
An applicable strategy for hit discovery. Docking-based high-throughput virtual screening can be used first to reduce the number of the molecule database, which can be evaluated by more accurate binding affinity calculations to optimize the docking results.
